# Correction: Keen et al. Establishing Innovative Complex Services: Learning from the Active Together Cancer Prehabilitation and Rehabilitation Service. *Healthcare* 2023, *11*, 3007

**DOI:** 10.3390/healthcare12121175

**Published:** 2024-06-11

**Authors:** Carol Keen, Gail Phillips, Michael Thelwell, Liam Humphreys, Laura Evans, Anna Myers, Gabriella Frith, Robert Copeland

**Affiliations:** 1 Sheffield Teaching Hospitals NHS Foundation Trust, Sheffield S9 3TY, UK; 2 Advanced Wellbeing Research Centre, Sheffield Hallam University, Sheffield S9 3TU, UK

## Addition of Two Authors

Anna Myers and Gabriella Frith were not included as authors in the original publication [1]. The corrected Author Contributions statement appears here. The authors state that the scientific conclusions are unaffected. This correction was approved by the Academic Editor. The original publication has also been updated.

**Author Contributions:** Conceptualisation, R.C., L.E., A.M. and C.K.; writing—original draft preparation, C.K. and R.C.; formal analysis—G.F.; writing—review and editing, C.K., R.C., L.E., A.M., G.F., G.P., L.H. and M.T. All authors have read and agreed to the published version of the manuscript.
